# Potentials of Interferons and Hydroxychloroquine for the Prophylaxis and Early Treatment of COVID-19

**DOI:** 10.33696/immunology.2.063

**Published:** 2020

**Authors:** Alexander Yang, Lakshmi S. Guduguntla, Bing Yang

**Affiliations:** 1 Center for Molecular Medicine and Genetics, School of Medicine, Wayne State University, 3939 Woodward Ave, Detroit, MI 48202, USA; 2 Wayne State School of Medicine, 540 E Canfield St, Detroit, MI 48201, USA; 3 Department of Biology, Saginaw Valley State University, 7400 Bay Rd, University Center, MI, USA

## Abstract

The symptoms of the COVID-19 range from asymptomatic or mild disease to severe disease that results in acute respiratory distress syndrome (ARDS) and eventually death. Understanding the molecular mechanisms responsible for the progression from mild to severe disease is the key to decreasing the mortality of COVID-19. Compared to mild cases, severe cases of the COVID-19 have decreased interferon (IFN) α, β, λ production. Type I (IFN α/β) and III IFNs (λ) work coordinately to induce inhibition of viral reproduction through the stimulation of interferon stimulated genes (ISGs). Failure to mount an IFN response leads to suboptimal activation of adaptive immune response and increased viral load. The increased viral load causes severe tissue damage, inducing a late wave of IFNs and an exacerbated inflammatory response. There are two known risk factors associated with severe disease- obesity and aging. Both lead to the activation of inflammasome NLRP3, which stimulates transcription factor NFκB and the production of inflammatory cytokines. Type I IFNs inhibit activation of NRLP3. Taken together, an early deficient IFN response and the following hyperinflammatory state are the hallmarks of severe COVID-19. This suggests that both type I and III IFNs could potentially be beneficial as prophylaxis and treatment of COVID-19 at the early stage of infection. Indeed, clinical studies have shown benefit of IFN Is, and there are ongoing trials testing type III IFNs for the treatment of COVID-19. Another strategy is to use hydroxychloroquine (HCQ) to inhibit the viral entry into the cells. Our reanalysis of the results from two randomized clinical trials (RCTs) has concluded that use of HCQ is beneficial in postexposure prophylaxis. These two strategies can have great potential in the current pandemic of COVID-19.

## Introduction

Severe acute respiratory syndrome coronavirus 2 (SARS-CoV2) causes a mild respiratory infection in most individuals. However, a portion of patients develop a severe infection resulting in the need for mechanical ventilation and ultimately death. Currently, the median infection fatality rate of coronavirus disease 2019 (COVID-19) is estimated to be 0.27% [1]. Understanding the molecular mechanisms underlying the progression of disease from mild to severe will lead to new therapies and prevent fatalities.

Severe cases of COVID-19 are marked by a hyperinflammatory response. The increased levels of proinflammatory cytokines lead to the accumulation and recruitment of leukocytes, resulting in acute respiratory distress syndrome (ARDS) [2]. From animal models and data from human samples, the lack or low production of IFNs at the early stage of infection and the unregulated inflammation at the late stage of the infection are hallmarks of severe disease. Thus, supplementing IFNs could be used for patients in the early stage of infection to prevent progression to severe disease.

We initially proposed to test the use of IFN α−2b together with HCQ for the prophylaxis of SARS-CoV-2 [3]. HCQ is known to inhibit endocytosis that is important for the viral entry into the cell [4]. In this review, we will review the current understanding about type I IFNs (IFN Is) and type III IFNs (IFN IIIs) in the pathogenesis of COVID-19 and summarize the current clinical data on the use of HCQ and IFNs for COVID-19.

## Inhibition of SARS-CoV-2 Entry into Cells by HCQ

SARS-CoV-2 enters the cell through the ACE2 receptor [5]. The S protein responsible for the entry needs to be primed by proteases that cleaves S into S1 and S2, resulting in attachment and membrane fusion. There are two mechanisms of S protein cleavage: endosomal cleavage through protease cathepsin B/L and plasma membrane protease TMPRSS2 cleavage. In both mechanisms, fusion with the membrane results in the release of viral nucleocapsid into the cytosol of the host cell [6].

**Figure 1:**
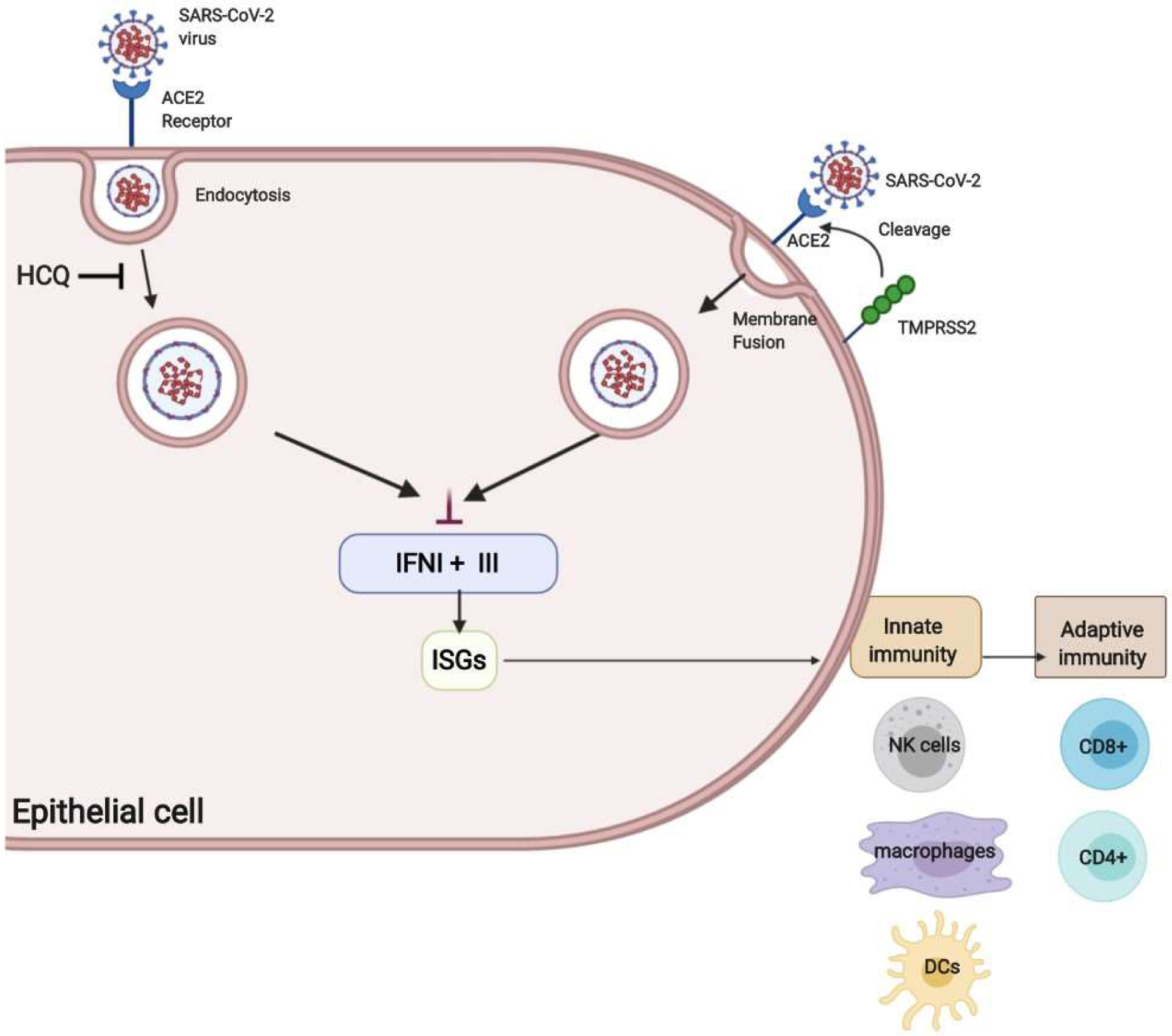
HCQ Inhibition of SARS-CoV-2 Entry and SARS-CoV-2 Inhibition of Interferon Response within Cell. Upon binding to the ACE2 receptor, SARS-CoV-2 can enter the cell through two different mechanisms-endocytosis and membrane fusion. Both are mediated by proteases that cleave the S protein. In endocytosis, cathepsin B/L cleavage occurs after endosomal acidification, and in membrane fusion, TMPRSS2 cleavage occurs on the surface on the cell. After entry into the cell, SARS-CoV-2 inhibits IFN I and IFN III response (IFN I +III), resulting in suppression of the adaptive immunity and increased viral load. HCQ has been shown to suppress endocytosis mediated entry into the cell.

HCQ has been shown to inhibit endocytosis-mediated viral entry *in vitro*. As a result, HCQ had high hopes to be effective in treatment and prophylaxis. However, evidence suggests that HCQ is not effective in treating the severe form of COVID-19. HCQ only blocks endosomal entry into the host cell and not TMPRSS2 mediated membrane fusion, thus limiting the effectiveness of HCQ inhibiting the SARS-CoV-2 entry into the host cell [6] (Figure 1). This could explain the mixed results of using HCQ for treatment of COVID-19 [7–12].

For prophylaxis, RCTs suggest HCQ is beneficial in post-exposure prophylaxis. A RCT conducted by Boulware et al. found no significant decrease in incidence of COVID-19 in individuals taking HCQ for post-exposure prophylaxis within four days of exposure compared to placebo (p=0.35) [13]. However, a preprint release without peer review suggested that the use of HCQ is time sensitive; earlier use provides increased protection (p=0.01) [14]. Our recent peer-reviewed publication also did a re-analysis of Boulware’s results using Cochran-Amitage analysis of trend. We found that HCQ offers protection against symptomatic COVID-19 (p=0.0496) in a time-dependent manner [15]. In another RCT study, Mitja et.al, found that postexposure use of HCQ resulted in a significant increase in SARS-CoV-2 IgG/IgM seroconversion compared to control. There was a 55.6% increase in the number of patients with IgM/IgG against the virus (P=0.01) in the HCQ group compared to placebo group on Day 14, suggesting early activation of adaptive immune response [16]. These results from RCTs support the use of HCQ for prophylaxis after the exposure to the virus. There are currently 60 clinical trials being conducted for the use of HCQ as prophylaxis, and more data should be available soon [17].

## IFN I Antiviral Functions

Discovered sixty years ago, IFNs are cytokines released by immune cells that were named for their ability to “interfere” with viral replication. They are divided into three classes: type I, II, and III based on receptor binding and functional activity. Unlike type I and III, type II only contains a single member, IFN-γ. Since type II IFNs appear unaffected in the response to COVID19, we will focus on IFN Is and III. Despite binding to different receptors, both IFN I and III signal through the JAK-STAT pathway. Activation of the JAK-STAT pathway results in translocation of IFN-stimulated gene factor 3 (ISGF3) complex to the nucleus resulting in upregulation of anti-viral interferon stimulated genes (ISGs) [18]. Transcriptome profiling of the ISGs shows shared gene signatures between IFN I and III, further adding to the similarities between the two classes of IFNs.

There are three types of IFN Is- IFN α, β, ω. Secreted IFNs I bind to IFN I receptors, which result in the assembly of the ISGF3 complex. ISGF3 activates the transcription of ISGs [18]. Type I ISGs inhibit viral protein translation, degrade and edit viral RNA, and inhibit viral RNA synthesis [19]. In addition, ISGs lead to the activation of NK cells, plasmacytoid dendritic cells, and macrophages to initiate the adaptive immunity [20]. The stimulation of IFN I release is mediated through the detection of pathogen-associated molecular patterns (PAMPs) and damage-associated molecular patterns (DAMPs) by pattern recognition receptors (PRRs). The secretion of IFN Is is mediated by two groups of PRRs- Toll-like receptors (TLRs) and RIG-I like helicases (RLHs). TLRs are located on the cytoplasmic surface and endosome where they recognize a wide range of PAMPs and DAMPs from lipoteichoic acid of Gram-positive bacteria to virus ssRNA. RLHs are localized to the cytosol and recognize virus dsDNA [21]. A more thorough review of the antiviral functions of IFN Is can be found from Charles Samuel [22].

IFN Is are important in the immune response against coronaviruses. Delayed activation of IFN I response to SARS-CoV-1 leads to increased pro-inflammatory cytokines, lung damage, and impaired T-cell response in both mice models [23] and human patients [24]. However, exogenous IFN I prior to viral peak load is protective against SARS CoV I [23]. Thus, the timing of IFN I secretion is crucial to consider when discussing IFN Is as a potential therapeutic for SARS-CoV-2.

## Role of IFN I in SARS-CoV-2

SARS-CoV-2 belongs to the virus family of coronaviruses and is a single-stranded, positive-sense RNA virus. Both TLR7/8 sense ssRNA sequences from viruses and are highly expressed in the endosomes of myeloid dendritic cells and monocytes. Bioinformatic analysis of the SARSCoV-2 genome revealed TLR7/8 recognition of large fragments of the viral genome, leading to induction of a IFN I response [25]. However, patients with severe SARS-CoV-2 instead show a delayed IFN I response and a poor immunological response against the virus.

In a study by Hadjadj et al., they examined the peripheral blood leukocytes in patients categorized as mild-moderate (n=15), severe (n=17), or critical (n=18). A decrease in all T cell subsets were found in severe and critical patients [21]. Further analysis of the transcriptional signature of peripheral white blood cells revealed significant decreases of IFN I in the blood, IFN I in the lung, and signature ISGs in critical patients compared to mild-moderate patients. This impaired IFN response was accompanied by increased NF-kB activation and increased expression of inflammatory genes including TNF and IL-6. IFN α2 protein levels in plasma were measured over a course of a week since the onset of symptoms. Mild-moderate patients had sustained high levels of IFN I; severe patients initially had high IFN I levels before a drastic decrease; and critical patients had sustained low levels of IFN I [26]. These results suggest that low levels of IFN I found in severe and critical patients lead to decreased T-cell response to SARS-CoV-2. Similar results were found by Blanco-Melo et al., in human bronchial epithelial cells and in ferrets infected with SARS-CoV-2 [27]. Taken together, these results suggest that a decreased IFN I response at the beginning of infection is a signature of severe COVID-19 and leads to progression of disease from mild to severe.

Lee, et al. performed single cell transcriptome analysis of peripheral blood mononuclear cells derived from mild and severe COVID-19 patients compared to patients with severe influenza. They found enrichment of IFN I ISGs along with TNF/IL-1β responsive genes in patients with severe influenza and severe COVID-19 in the late stage of the disease. Patients with mild COVID-19 were lacking in IFN I response genes. To further validate their study, they performed RNA-seq analysis on post-mortem lung tissues and classical monocytes from patients that passed away from severe COVID-19. IFN I and TNF/IL-1β response genes were upregulated in these tissues as well [28].

The increase in IFN I ISGs found in severe COVID-19 patients from Lee, et al. reflects a delayed activation of IFN I response [28]. The lack of IFN I in the early stage of the virus leads to unchecked viral replication, increased viral load, and cellular damage. Viral RNA and DAMPs from infected cells are sensed by TLRs, generating a delayed wave of IFN-I. Indeed, Arunachalam et al. measured the IFN α of 40 patients, and patients with moderate symptoms produce an IFN α peak 10 days after the onset of symptoms. However, patients with severe symptoms and in critical care have elevated IFN α peaks between 10–20 days [29]. Taken together, a delayed IFN I response leads to unchecked viral replication and progression to severe COVID-19.

## Effect of Aging and Obesity on Mortality of COVID-19

**Figure 2:**
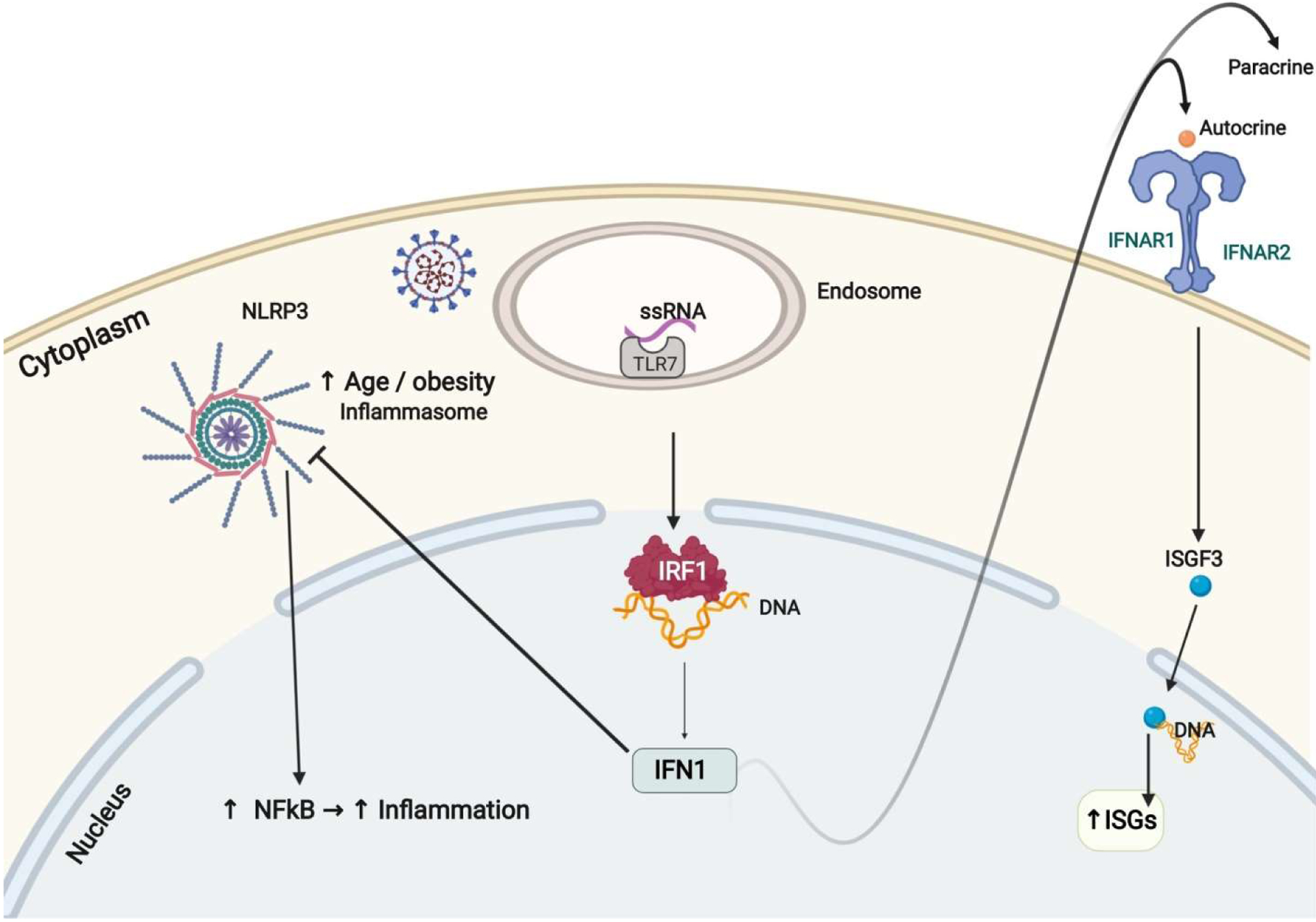
Type I IFN Response against SARS-CoV-2. After SARS-CoV2 entry into the host cell, TLR7 detects the ssRNA from the virus, activating interferon regulatory factor 1 (IRF1). IRF1 translocation to the nucleus results IFN I production. IFN I binds to heterodimeric transmembrane receptor composed of IFNAR1 and IFNAR2 subunits, acting in both autocrine and paracrine fashion. After binding to the receptor, activation of ISGF3 results in translation to the nucleus resulting in increased expression of ISGs, which then activate the anti-viral state of the cell. IFN Is also inhibit NLRP3, the inflammasome leading to decreased NFkB activation and inflammation.

Aging and obesity are the two main risk factors for severe COVID-19. The central player identified in predisposing aging and obese patients to cytokine storm is NLRP3, an inflammasome that activates NFκB, generating a pro-inflammatory cytokine response [24]. The activity of NLRP3 is under direct control by sirtuin 2 (SIRT2). In older individuals over 65, a decrease in NAD+ levels reduces the activity of SIRT2, leading to hyperactivation of NLRP3 and increased cytokine storms found in older COVID-19 patients [30]. In addition, obesity has been found to increase the activity of NLRP3 in mice, explaining the increased mortality in obese patients as well [31]. Interestingly, IFN Is have been implicated in counter-regulating NLRP3. IFN Is stimulate NO production and suppress ROS generation leading to suppression of NLRP3 activation [32,33]. Low or absent levels of IFN I lead to the unchecked activation of NLRP3, further predisposing elder and obese patients to severe form of COVID-19. In addition to activating the innate and adaptive immune system, IFN I suppression of NLRP3 is another therapeutic mechanism against COVID-19 (Figure 2).

## IFN for the Treatment for COVID-19

The 3a protein of SARS-CoV-1 virus has been shown to increase ER stress, resulting in the downregulation of IFN I receptor expression [34]. However, this is not the case for SARS-CoV-2. Hadjadj et al. have found that there is a slight increase of expression of IFN I receptor expression in mild-moderate patients and a drastic increase of expression in severe and critical patients of SARS-CoV-2 [26]. Arunachalam et al. did not report any change of IFN I receptor expression but found an increased expression of ISGs in patients with COVID-19 in his patient group [29]. The intact expression and functionality of IFN I receptor found in patients infected with SARS-CoV-2 suggests that IFN I is a viable therapeutic option for SARS-CoV-2.

Recently, a 4-nucleotide loss of function deletion and a predicted loss of function missense variant in TLR7 were identified in four individuals from two different families that were admitted to the ICU due to severe COVID-19 [35]. All four required mechanical ventilation for an average of 10 days and one patient died. PMBCs isolated from the patients resulted in decreased IFN I ISGs upon stimulation of TLR7 compared to healthy controls. Deficient IFN I levels and loss of function TLR7 variants correlated with severe COVID-19 suggest the use of IFN I to prevent progression of disease.

In a multicenter, prospective, open label, randomized trial from six hospitals in Hong Kong, triple antiviral therapy lopinavir-ritonavir, ribavirin subcutaneous injection of 8 million IU interferon beta-1b was compared to lopinavir-ritonavir alone [36]. Patients in the triple antiviral therapy group had a significantly shorter course of the virus (7 days) compared to lopinaivir-ritonavir alone (12 days), as detected by RT-PCR from nasopharyngeal swab viral load. As a result of the shortened course of the virus, the overall hospital stay time was shortened to 9 days compared to two-drug combination therapy as well, 14.5 days.

In a press release by Synairgen PLC, inhaled IFN beta showed 79% reduction in risk of developing severe COVID-19 compared to placebo [37]. Another preprint study done in Wuhan, China found nebulized IFN-α2b significantly reduced duration of detectable virus and inflammatory markers IL-6 and CRP compared to treatment with antiviral arbidol alone [38]. Taken together, these clinical trials combined with the deficient IFN I signature found in severe patients suggest the efficacy of IFN I as a part of an antiviral therapy for COVID-19.

## Antiviral Functions of Type III Interferons

Like IFN Is, IFN IIIs, or IFN λ are activated upon PRR recognition of PAMPs and DAMPs, and signal through the JAK-STAT pathway. Unlike the ubiquitous expressed IFN I receptors, IFN III receptors are expressed preferentially on epithelial cells [39]. While both IFN I and IFN III ISGs overlap, type IFN III signaling results in more prolonged expression of ISGs. In addition, only IFN I signaling results in pro-inflammatory cytokine release [40]. Mice that lack receptors for both IFN I and IFN III are more prone to respiratory infections such as influenza and SARS-CoV-1 compared to IFN I receptor deficient mice alone, suggesting a protective function of IFN IIIs in the epithelium of the respiratory system [39]. However, unlike IFN Is, IFN IIIs have not yet been approved for any antiviral therapies.

In addition to a deficient IFN I, Blanco-Melo et al., also found IFN III deficiency in COVID-19 patients [27]. *In vitro*, recombinant human IFN λ inhibited viral replication in a dose-dependent manner in Calu-3 and Vero E6 cells [41]. Taken together, these results suggest that like IFN I, IFN III could be beneficial in the treatment for COVID-19. Due to the localized expression of IFN III receptors on epithelial cells and the lack of inflammatory cytokine release, IFN III could potentially offer the same benefits of IFN I treatment without the risk of inducing a hyper-inflammatory response.

However, in an animal study, IFN λ disrupts the lung epithelial barrier, resulting in lethal bacterial infection [42]. Thus, the safety of IFN III in the prophylaxis and treatment of COVID-19 is still being investigated with two current ongoing clinical trials. One clinical trial that is still recruiting is a randomized, double-blind, placebo-controlled trial of 140 participants from the University Health Network of Toronto. The study aims to explore the impact of pegylated IFN λ in an ambulatory cohort of relatively healthy COVID positive patients who can tolerate home isolation. The effectiveness of pegylated IFN λ compared to a saline placebo will be evaluated by measuring the proportion of SARS-CoV-2 negative patients on day 3 and then on day 7 of the treatment. This trial is an adaptive study that will be applied to a hospitalized cohort after the initial assessment of the response and safety of the treatment. Another clinical trial underway from Johns Hopkins University with an estimated target enrollment of 164 participants. The trial will measure the proportion of participants with no evidence of SARS-CoV-2 infection for 28 days. The results from these studies will hopefully shed light on the feasibility of IFN λ as a potential treatment for the SARS-CoV-2 virus.

## Conclusions

In the mild form of COVID-19, IFN I and III activation of the innate and adaptive immune response prevents progression of disease. In contrast, patients with severe COVID-19 have low production of IFN I and III leading to decreased activation of the adaptive immunity. The delayed activation of adaptive immune response results in lymphopenia and unchecked viral replication. The increased tissue damage from the virus induces pro-inflammatory cytokines and a second wave of IFN I, leading to cytokine storm and ultimately death. Although initial results are promising, the therapeutic benefits of IFN I and III against COVID-19 are still being investigated in clinical trials.
